# Questionnaire-based real-world survey of diagnosing food allergy in children: Utilization of oral food challenge tests and other diagnostic methods

**DOI:** 10.1016/j.jacig.2024.100356

**Published:** 2024-10-18

**Authors:** Chisa Kumagai, Norio Kawamoto, Yuki Miwa, Tomoko Kaneyama, Saori Kadowaki, Minako Kawamoto, Hidenori Ohnishi

**Affiliations:** a Department of Pediatrics, Graduate School of Medicine, Gifu University, Gifu, Japan; b Allergy Center, Gifu University Hospital, Gifu, Japan

**Keywords:** Anaphylaxis, food allergy, Japan, patient care management, surveys, questionnaires

## Abstract

**Background:**

Oral food challenge tests are considered the reference standard for diagnosing food allergies; however, studies on their real-world implementation rates are limited.

**Objective:**

The study aimed to investigate the proportion of school-age children who underwent the oral food challenge test and to understand the motivations behind food elimination and utilization of various health care services.

**Methods:**

The questionnaire-based survey for the parents of the students who submitted the “Certificate for School Life Management (For Allergic Diseases)” was conducted across public elementary and junior high schools in Gifu prefecture, Japan.

**Results:**

The study encompassed parents of 3457 children with food allergies who submitted the certificate. Approximately one third of those eliminating the 3 major allergens—eggs (32.5%), milk (27.6%), and wheat (33.5%)—were diagnosed via oral food challenge tests, and approximately two thirds were diagnosed using a combination of symptoms and blood tests, suggesting most children were diagnosed appropriately. However, many children were diagnosed and eliminated foods based solely on blood tests without any symptoms of other allergens, such as buckwheat (55.8%), peanuts (29.2%), and tree nuts (21.2%), suggesting that it was likely that these children unnecessarily eliminated foods. Elimination of buckwheat because of anxiety was associated with eliminating other foods for the same reason and with eliminating 2 or more foods.

**Conclusion:**

Examination of the real-world application of the proposed recommendations for the accurate diagnosis of food allergies suggests that closely monitoring their practical application should be conducted in each case to avoid unnecessary food elimination from children’s diets.

Food allergy, a common disease in children, is defined as adverse reactions to food caused by antigen-specific immunologic mechanisms. It affects 9.3% and 7.6% of children aged ≤18 years in Europe and the United States, respectively, and 4.5% of school-age children in Japan.^1^, ^2^, ^3^ Food allergy can precipitate anaphylaxis, the most severe clinical presentation of acute systemic allergic reactions, leading to impairment in the quality of life. Given that children with food allergies spend a significant portion of their time in school, where they are provided with school lunches, they require special care at school.

Food allergies are diagnosed by detailed history, immunologic examination, and oral food challenge (OFC) test.^3^, ^4^, ^5^, ^6^ The basic management of food allergy involves eliminating the diagnosed causative foods. The Japanese food allergy guidelines recommend “minimum avoidance of causative foods” based on the appropriate diagnosis; thus, caution should be exercised in not eliminating causative foods excessively.^3^ Extensive and long-term food elimination can cause nutritional deficiencies and quality of life impairment.^7^^,^^8^ OFC is the most reliable method for identifying the causative foods and is recommended for obtaining a definitive diagnosis or confirming the acquisition of tolerance.^3^, ^4^, ^5^, ^6^^,^^9^ Previous reports have demonstrated that OFC improves the quality of life of children with food allergies and their parents.^10^^,^^11^ A mixed-methods study involving allergists, pediatricians, and parents of patients with food allergies in Canada revealed that resource scarcity is a barrier for allergists, and the pediatricians complained of long wait times, highlighting the insufficient implementation of OFC.^12^ Greiwe et al showed that 63.5% of allergists in the United States performed only 5 or fewer OFCs per month as a result of lack of time and human resources.^13^ A small island-wide survey in Japan reported that 55% of children who restricted their intake of certain foods suspected to cause food allergies had unnecessarily eliminated foods.^14^ Although previous studies have shown that OFC may not be appropriately implemented in the management of food allergies,^12^, ^13^, ^14^ to our knowledge, no study has yet investigated the rate of OFC implementation for each culprit or allergenic food.

The study aimed to investigate the proportion of schoolchildren who underwent OFC test and the reasons for food elimination using an allergy survey among school-age children of public elementary and junior high schools in Gifu prefecture, a local region of Japan. We postulated that clarification on this topic would facilitate improved management of food allergies.

## Methods

### Study design and participants

This study was performed using datasets from a prefecture-wide survey on the real-life diagnosis, management, and parental perceptions of allergic diseases in schoolchildren conducted in Gifu prefecture, Japan, in 2019. Given that 99% of elementary and junior high school students in Gifu prefecture attend public schools, this survey encompassed almost all parents of elementary and junior high school students in Gifu prefecture.^15^^,^^16^

Because this survey was conducted with the support of the Gifu Prefectural Board of Education, permission was obtained from all public schools. The first to sixth grades of compulsory education schools and special needs schools correspond to elementary school, and the students were considered as elementary school students in this study. Furthermore, seventh to ninth grades correspond to junior high school, so such students were considered to be junior high school students in this study. At the time of the survey, Gifu prefecture had 367 elementary schools, 176 junior high schools, 2 combined elementary and junior high schools, and 20 special needs schools.^16^

Children with allergic diseases who require allergy support at school are required to submit a “Certificate for School Life Management (For Allergic Diseases).” In particular, patients who require an elimination diet for their school lunch must submit these certificates. Therefore, it is presumed that most children with food allergies submit this report. The parents of the students who submitted this certificate in public elementary and junior high schools in Gifu prefecture were recruited for this study. This certificate, filled out by the attending physician or school physician, provides information on the child’s allergic diseases and is submitted to the school via the parents. The information on food allergies includes the clinical type, presence or absence of anaphylaxis, causative food and reason for elimination, prescription medications in case of emergency, and whether special considerations for school lunches are required. Schools should determine how to manage each student with an allergic disease by consulting with their parents on the basis of the certificates. According to a Gifu Prefectural Board of Education survey in the same year, 107,378 elementary school students and 55,796 junior high school students were enrolled in public schools, of which 3526 elementary school students and 1679 junior high school students submitted certificates.

The questionnaires were distributed by the school nurses at each school to the students who had submitted the certificate, and parents willing to participate in this study received the questionnaires from their children and filled them out. Questionnaires were administered in cooperation with the Gifu Prefectural Board of Education. This study was approved by the ethics committee of the Gifu University Graduate School of Medicine (approval 2019-056). Written informed consent for participation in the survey was obtained from the parents of the children.

### Questionnaire

The survey included the following questionnaire items: school district, school grade, presence or absence of each allergic disease (food allergies, atopic dermatitis, bronchial asthma, allergic rhinitis, and allergic conjunctivitis), clinical types of food allergies, history of anaphylaxis, and cause of anaphylaxis. The questions were structured in a manner analogous to the “Certificate for School Life Management.” Additionally, the questionnaire included items pertaining to the primary care physicians consulted by the parents regarding their children’s food allergies, the prescription of adrenaline auto-injectors, the type of foods eliminated, and the rationales leading to food elimination.

To determine the number and type of food items eliminated from the diet of a particular child, food categories were chosen from the following options (eggs, milk, wheat, buckwheat, peanuts, tree nuts, fruits, fish, meat, crustaceans, and others). To determine the clinical type of food allergies in children, parents responded to a multiple-choice question inquiring about their medical condition (immediate-type food allergy, oral allergy syndrome, and food-dependent exercise-induced anaphylaxis) from a set of 3 possible responses (yes, no, and unknown). To determine how a particular food was deemed an allergen for their children, parents selected 1 of the following 5 options:

1. The child had obvious symptoms and was diagnosed with an allergy by a physician with a blood test.
2. The child experienced allergic symptoms, allergy was confirmed by blood tests, and an OFC test was performed by a physician, resulting in a diagnosis.
3. The child had a positive blood test result but has never consumed the food.
4. The child experienced allergic symptoms but had no blood tests or OFC performed, and was not diagnosed by a doctor.
5. Parents or children were worried about allergic symptoms and avoided those particular foods at their own discretion.

### Data analysis

A survey was conducted using a bubble answer sheet. Questionnaire data were digitized using a scanning program for bubble answer sheets, and each input value was confirmed by the research team. For each question, incorrect answers, such as multiple answers to a question requiring a single answer, were excluded from the analysis.

Calculations and graphs were generated by Microsoft Excel 2019 (Microsoft, Redmond, Wash). The Fisher exact test was performed by R software (R Project; www.r-project.org). *P* < .05 was considered statistically significant.

## Results

### Characteristics of study participants

**Fig 1:**
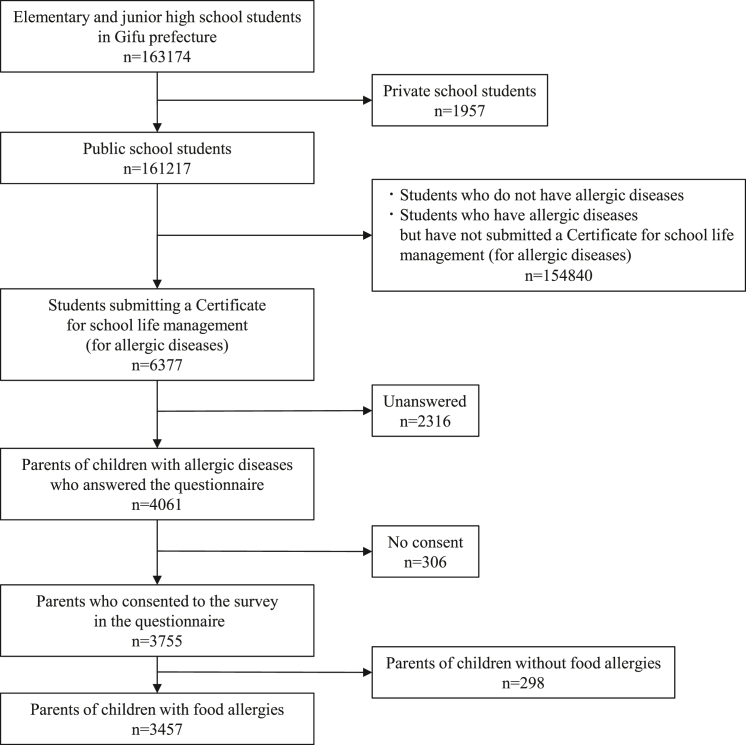
Study flow diagram.

Among the 6377 students who submitted the “Certificate for School Life Management,” the parents of 3755 students, comprising 2686 elementary school students and 1067 junior high school students, provided written consent to participate in this study (Fig 1, Table I). The number of respondents across each school grade is shown in Table E1 in this article’s Online Repository available at www.jaci-global.org. Of the 3457 students who had a food allergy, 2066 had an immediate-type food allergy, 1314 had an oral allergy syndrome, and 252 experienced food-dependent exercise-induced anaphylaxis (Table I).

**Table I:** Characteristics of children with food allergies

Characteristic	Elementary school	Junior high school	Unknown∗	Total
Total	2686	1067	2	3755
Food allergy†	2470	986	1	3457
Immediate type	1523	543	—	2066
OAS	859	455	—	1314
FDEIA	152	100	—	252

*FDEIA,* Food-dependent, exercise-induced anaphylaxis; *OAS,* oral allergy syndrome.

∗ Unknown represents study participants (parents) who did not appropriately respond to question concerning their children’s school grades.

† Children with food allergies categorized into the clinical type include multiple responses.

Parents were queried about the type of primary care physicians that they consulted for their children’s food allergies. Children with allergic diseases visited various medical facilities (Table II). Most children with food allergies had a hospital pediatrician as the primary physician (50.3%). Some patients mainly visited dermatologists and otolaryngologists in clinics (3.3% and 3.4%, respectively).

**Table II:** Primary physicians consulted for food allergies

Physician discipline	Clinic, no. (%)	Hospital, no. (%)
Internal medicine	251 (8.0)	158 (5.1)
Pediatrics	766 (24.5)	1573 (50.3)
Otorhinolaryngology	102 (3.3)	69 (2.2)
Dermatology	105 (3.4)	102 (3.3)
Ophthalmology	1 (0.0)	0 (0.0)

### Food elimination

**Fig 2:**
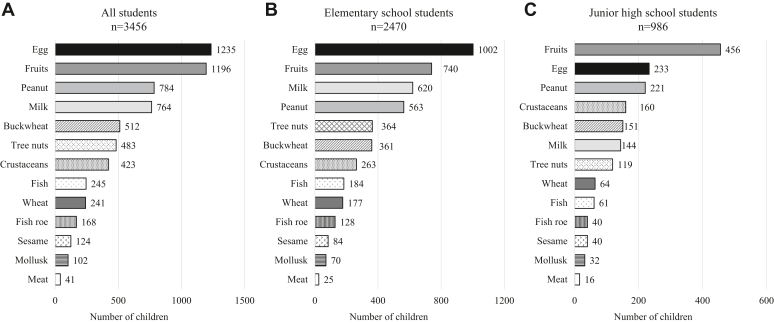
Specific food items eliminated from diets of children with food allergies. **(A)** Entire study population. **(B)** Elementary school children. **(C)** Junior high school children. Numbers in each bar indicate number of students who eliminated each food item. Multiple responses were collected; some students eliminated more than 1 food item from their diets.

Fig 2 shows the foods eliminated by elementary and junior high school students in descending order: eggs (n = 1235), followed by fruit (n = 1196), peanuts (n = 784), milk (n = 764), and buckwheat (n = 512) (Fig 2, *A*). The most commonly eliminated foods in the diets of elementary school students in the study group were eggs (n = 1002), fruits (n = 740), milk (n = 620), peanuts (n = 563), and tree nuts (n = 364) (Fig 2, *B*). In contrast, among the junior high school students, fruits (n = 456) were frequently eliminated, followed by eggs (n = 233), peanuts (n = 221), and crustaceans (n = 160) (Fig 2, *C*).

**Fig 3:**
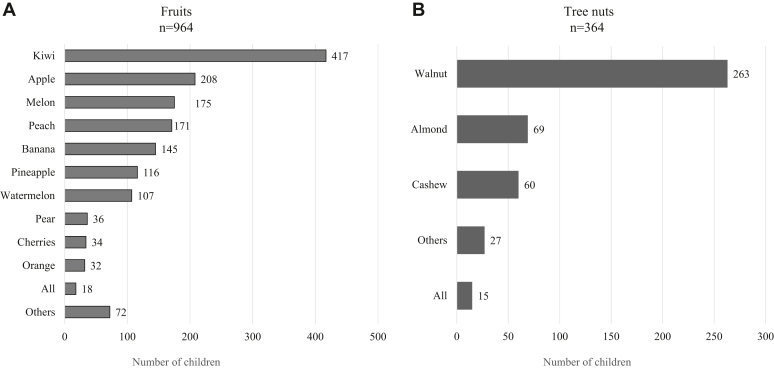
Dietary exclusions of fruits and tree nuts among children with food allergies. Number of schoolchildren who eliminated **(A)** fruits and **(B)** tree nuts from their diets. Children who had to eliminate all tree nuts or fruits were categorized under “all.” Each bar represents specific food item; numerical value within it indicates number of students who eliminated that item from their diet.

Among the fruits, kiwi (n = 417) was the most common fruit eliminated. Many students also eliminated Rosaceae fruits such as apples and peaches (n = 208 and n = 171, respectively) (Fig 3, *A*). The tree nuts that most students eliminated were walnuts (n = 263), followed by almonds (n = 69) (Fig 3, *B*).

**Fig 4:**
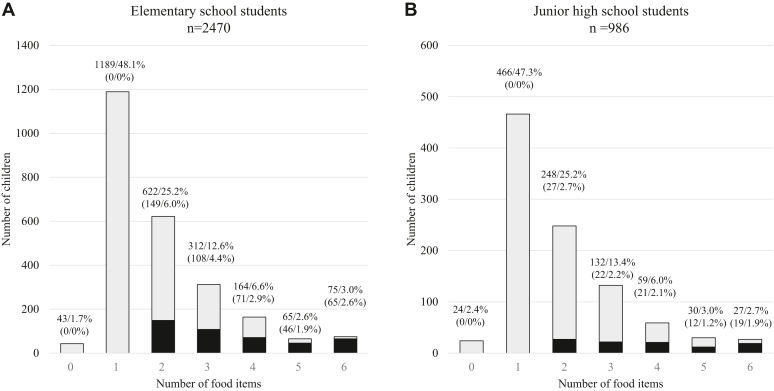
Number of food items eliminated from diets of children with food allergies. **(A)** Elementary school students. **(B)** Junior high school students. *Black bars* indicate number of children who eliminated at least 2 items from eggs, milk, or wheat. Numbers on bars indicate numbers of students eliminating particular foods and percentages of children with food allergies in population; numbers in parentheses indicate numbers and percentages of students who eliminated 2 or more food items, particularly eggs, milk, or wheat.

We also analyzed the number of foods eliminated per student (Fig 4). Among elementary and junior high school students, approximately half of the children with food allergies eliminated only 1 food item. In contrast, the other half eliminated multiple foods; 75 elementary and 27 junior high school students eliminated 6 types of food. In addition, 15.6% (n = 540) of the children with food allergies eliminated multiple foods, including eggs, milk, and wheat.

### Reason for eliminating foods

**Fig 5:**
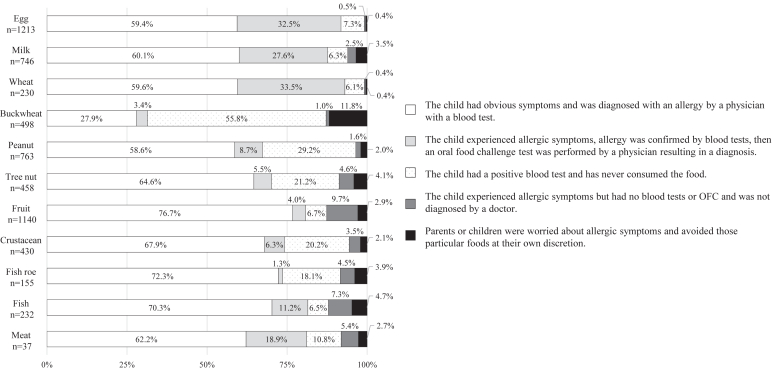
Motivations for food elimination from children’s diets according to parental responses. Stratification of diagnostic evidence-based reasons for avoiding consumption of certain foods by children with particular food allergies was based on survey responses of parents. Number of children who eliminated each food item is shown under each item on the y-axis.

To investigate the underlying rationale for food allergy diagnosis and the prevalence of OFC testing for each food item, we inquired about motivations for eliminating food (Fig 5). Owing to obvious symptoms and positive blood test results, 59.4%, 60.1%, and 59.6% of children eliminated eggs, milk, and wheat, respectively. Furthermore, owing to a diagnosis that was based on OFC, 32.5%, 27.6%, and 33.5% of the children eliminated eggs, milk, and wheat, respectively. A small percentage of participants responded that the child had a positive blood test result and had never consumed the 3 major allergens in Japan: eggs (7.3%), milk (6.3%), and wheat (6.1%). However, this pattern was not observed for other allergens, such as buckwheat, peanuts, tree nuts, crustaceans, and fish roe. Among the children who eliminated buckwheat, 55.8% had not consumed it but eliminated it as a result of a positive blood test result, and 11.8% eliminated it because of anxiety despite not having a physician’s diagnosis—a trend that was more pronounced for buckwheat than for other foods. Regarding peanuts, tree nuts, crustaceans, and fish roe, 29.2%, 21.2%, 20.2%, and 18.1% of the children, respectively, eliminated them as a result of a positive blood test result and had never consumed them.

Given that self-imposed food elimination is not considered appropriate, we focused on the background of those who chose option 5, “parents or children were worried about allergic symptoms and avoided those particular foods at their own discretion.” We analyzed 498 participants who responded to the question on buckwheat elimination. Of these, 59 (11.8%) were eliminating buckwheat because of anxiety without any symptoms, blood tests, or OFC. Eliminating buckwheat because of anxiety was associated with the elimination of eggs, milk, peanuts, and tree nuts for the same reason, as well as the elimination of 2 or more foods (*P* = .004, *P* = .023, *P* < .001, *P* = .002, and *P* = .046, respectively) (Table III). However, the reasons for eliminating buckwheat were not associated with anaphylaxis, bronchial asthma, atopic dermatitis, or type of medical institution attended.

**Table III:** Characteristics of children who eliminated buckwheat from their diets

Characteristic	Eliminated because of anxiety∗	Eliminated for other reasons	*P* value
Immediate food allergy	48/50 (96.0%)	298/312 (95.5%)	1.000
Anaphylaxis caused by some foods	28/36 (77.8%)	212/265 (80.0%)	.825
Bronchial asthma	18/55 (32.7%)	119/405 (29.4%)	.638
Atopic dermatitis	26/57 (45.6%)	208/411 (50.6%)	.572
Medical institutions attended for food allergies			
Clinic	15/57 (26.3%)	134/415 (32.3%)	.448
Hospital	42/57 (73.7%)	281/415 (67.7%)	.448
Not pediatricians	8/57 (14.0%)	96/415 (23.1%)	.129
Children with adrenaline auto-injectors	16/56 (28.6%)	128/426 (30.0%)	.878
Foods eliminated because of anxiety			
Eggs	3/40 (7.5%)	0/218 (0.0%)	.004
Milk	3/23 (13.0%)	2/134 (1.5%)	.023
Wheat	1/10 (10.0%)	0/90 (0.0%)	.096
Peanuts	10/27 (37.0%)	2/221 (0.9%)	<.001
Tree nuts	4/11 (36.4%)	4/111 (3.6%)	.002
Fruits	2/14 (14.3%)	6/172 (3.5%)	.114
No. of foods eliminated			
Two or more	58/59 (98.3%)	398/439 (90.7%)	.046
Three or more	47/59 (79.7%)	311/439 (70.8%)	.169
Four or more	25/59 (42.4%)	220/439 (50.1%)	.271
Five or more	15/59 (25.4%)	129/439 (29.4%)	.647
Six or more	9/59 (15.3%)	71/439 (16.2%)	1.000

∗ Children of parents who chose option “parents or children were worried about allergic symptoms and avoided those particular foods at their own discretion” were defined as those eliminating buckwheat because of anxiety.

## Discussion

In this study, we investigated the proportion of school-age children with allergies who underwent OFC testing and their motivations for food elimination, thereby highlighting the reality of food elimination in this demographic with food allergies. Among these children, approximately one third of those who eliminated the 3 major allergens in Japan (eggs, milk, and wheat) were diagnosed by OFC, and approximately two thirds were diagnosed using a combination of apparent symptoms and blood tests, suggesting that the majority of diagnoses were conducted appropriately. However, a significant number of children who eliminated other allergens, such as buckwheat, peanuts, and tree nuts, were diagnosed only with blood tests, suggesting that there were many children who unnecessarily eliminated foods for fear of an allergic reaction.

The 2020 Japanese guideline for food allergy stipulates that the principle of food allergy management is “minimum avoidance of causative foods based on correct diagnosis.”^3^ Our study population might include inappropriate elimination for managing allergens such as buckwheat, peanuts, and tree nuts. More than half of the study participants eliminated buckwheat on the basis of blood test results, without any history of allergic reactions. Buckwheat is the ninth most common major antigen, and the incidence of immediate type food allergies was 1.8% in Japan.^17^ Furthermore, buckwheat is the fourth most common food that can cause anaphylactic shock (16.5%).^17^ Yanagida et al reported that only 8.1% of patients positive for buckwheat-specific IgE antibodies in their serum and no history of buckwheat-related allergic reaction had a positive OFC result.^18^ In this study, 55.8% of the students eliminated buckwheat on the basis of blood test results only, without experiencing symptoms. Our results suggest that a considerable number of children in our study unnecessarily eliminated buckwheat from their diets, which indicates the need for a confirmatory diagnosis to prevent unwarranted food elimination.

More than 1 in 5 participants eliminated peanuts and tree nuts solely on the basis of blood test results, without any history of allergic reactions to peanuts or tree nuts. These foods are less commonly consumed than eggs and milk but are often associated with anaphylaxis. Cashews and walnuts were found to be the first and third most common foods causing anaphylactic shock (18.3% and 16.7%, respectively).^17^ In contrast, tree nuts and peanuts were the fourth and fifth major allergens, and the incidences of immediate-type food allergies were 8.2% and 5.1%, respectively.^17^ Recently the importance of component-based diagnosis of walnuts and cashews has been reported.^19^, ^20^, ^21^, ^22^ The allergen components of nuts, such as Jug r 1 of walnut and Ana o 3 of cashew, were covered by Japanese public insurance in 2018, implemented 6 months before our study was conducted; consequently, we found that many children eliminated tree nuts according to their blood test results but without having a history of allergic reactions, resulting in unnecessary elimination of tree nuts from their diet. Ideally, these children should be accurately diagnosed by OFC test because the incidence of immediate-type food allergies induced by tree nuts increased from 2.3% in 2011 to 8.2% in 2018.^17^^,^^23^

In contrast to allergens such as buckwheat, peanuts, and tree nuts, most study participants eliminated 3 major allergens—eggs, milk, and wheat—on the basis of a physician’s diagnosis; approximately one third of those eliminating these allergens (32.5%, 27.6%, and 33.5%, respectively) were diagnosed by OFC test, and approximately two thirds (59.4%, 60.1%, 59.6%, respectively) were diagnosed on the basis of obvious symptoms and blood tests. Less than 5% of the children eliminated these major allergens from their diet without a doctor’s diagnosis. A survey reported by Kawaguchi et al, conducted among patients who visited a specialized tertiary-care hospital for food allergies, found that most patients older than 6 eliminated certain foods, such as eggs, milk, and wheat, on the basis of a physician’s diagnosis.^24^ In contrast, Okada et al reported that 55% of children who were suspected of having a food allergy were able to eat those foods with an OFC-based diagnosis provided by a doctor, and they reported that unnecessary elimination of eggs and milk is most commonly found among children.^14^ In addition, a study found that only one third of children with food allergies were evaluated by OFC testing, even for the 3 major allergens, although OFC is widely used in Japan.^25^ These findings suggest that in a real-world setting, including areas without easy access to specialized medical care, there are children who do not receive adequate medical care for food allergies, even for major allergens.

The elimination of multiple foods can affect the weight of children.^26^^,^^27^ Therefore, it is necessary to facilitate a more precise diagnosis of food allergies in such children. In our study, approximately one fourth of all the students eliminated 3 or more foods because of food allergies. Stensgaard et al and DunnGalvin et al reported that the number of foods eliminated caused greater impairment in quality of life.^28^^,^^29^ Among the children who eliminated buckwheat in our study, more than half had not consumed it but eliminated it because of a positive blood test result, and at least 1 in 10 eliminated it owing to anxiety. Although the causes of anxiety were not directly investigated, the elimination of buckwheat because of anxiety was associated with the elimination of other foods for the same reason, but not with comorbidities, types of medical care received, or number of foods eliminated. These children could be relieved from eliminating some foods if they received appropriate medical care.

The number of patients who needed to eliminate eggs, fruits, peanuts, and milk, followed by buckwheat and tree nuts, was high in our study. The order differs from the major allergens in Japan, which are based on visits to medical institutions.^17^ Our results are more likely to show the real prevalence of food allergies, including patients who previously had symptoms and who never again consumed the same culprit food. Further, several children who eliminate fruits have oral allergy syndrome. However, this study did not determine the clinical type of each food; therefore, further investigation is needed.

A characteristic of Japanese medical services is the freedom of choice of medical institutions or free access backed by universal health coverage.^30^ The patients can choose various types of institutes and a variety of specialists from each institute. The participants in our study visited various medical institutions, which may be attributed to the fact that the survey was conducted in cooperation with schools. Therefore, the results of this study provide information about children receiving various types of medical care and are not limited to specialty hospitals.

Our study has several advantages. First, it included parents of students attending public schools throughout the Gifu prefecture who had submitted a “Certificate for School Life Management,” implying inclusion of a more comprehensive range of people with allergic diseases than only those visiting specific medical institutions. Second, a more realistic evaluation of the appropriateness of such actions could be made by investigating the detailed reasons for eliminating certain foods from the perspective of guardians rather than physicians. Specifically, our study is the first large-scale general population survey to investigate the proportion of food allergies diagnosed using OFC for each food item.

Our study has several limitations. First, because this was a survey of students who had submitted the “Certificate for School Life Management,” children who did not submit a certificate (ie, children with food allergies to foods not provided in the school lunch) were most likely excluded. Second, there may have been discrepancies between the children’s diagnoses reported by the parents versus their actual medical records. Parents were asked to answer questions according to information provided in the certificates; however, recall bias may have affected the results. Finally, it should be noted that this is a real-world study that is not limited to diagnoses made by pediatric allergists in terms of diagnostic accuracy.

In conclusion, our study is the first large-scale general population survey to investigate the proportion of schoolchildren with food allergies diagnosed using OFC for each food item. Our study highlights the need for more accurate diagnosis and management of food allergies, which are common in school-age children. Our findings could help health care systems and schools to better allocate resources for allergy management as well as prevent unwarranted food elimination.

**Key messages**

• Most study participants eliminated 3 major allergens—eggs, milk, and wheat—on the basis of a physician’s diagnosis; approximately one third of these were diagnosed by OFC, and approximately two thirds were diagnosed on the bases of obvious symptoms and blood tests.
• Less than 5% of the children eliminated the 3 major allergens from their diet without a doctor’s diagnosis.
• Two-thirds of children unnecessarily eliminated buckwheat from their diets, without any allergy history, indicating the need for a confirmatory diagnosis to prevent unwarranted food elimination in schools to better allocate resources for allergy management.

## Disclosure statement

Commissioned by Gifu prefecture.

Disclosure of potential conflict of interest: The authors declare that they have no relevant conflicts of interest.

## Supplementary data

Supplementary Table E1
