# Supplementary material for: Questionnaire-based real-world survey of diagnosing food allergy in children: Utilization of oral food challenge tests and other diagnostic methods
Source: J Allergy Clin Immunol Glob. 2024 Oct 18;4(1):100356. doi: 10.1016/j.jacig.2024.100356 (PMC11585707; doi:10.1016/j.jacig.2024.100356)
Supplement: Supplementary Table E1 [file mmc1.docx]

**Supplementary Table Ⅰ.** Number of children with food allergies surveyed per grade

|  | **Number of the students^*^** | **Children with**  **food allergy** |
| --- | --- | --- |
| Total | 160072 | 3457 |
| Elementary school students | 106164 | 2470 |
| 1 | 16957 | 467 |
| 2 | 17411 | 433 |
| 3 | 17711 | 421 |
| 4 | 17607 | 375 |
| 5 | 18223 | 403 |
| 6 | 18255 | 340 |
| Unknown | － | 31 |
| Junior high school students | 53908 | 986 |
| 7 | 18045 | 363 |
| 8 | 17659 | 301 |
| 9 | 18204 | 295 |
| Unknown | － | 27 |

^*^The number of students at that school grade
